# Vitamin D supplementation for improving sperm parameters in infertile men: A systematic review and meta-analysis of randomized clinical trials

**DOI:** 10.1080/2090598X.2023.2165232

**Published:** 2023-01-10

**Authors:** Clarissa Tania, Edwin Raja Pardamean Lumban Tobing, Christiano Tansol, Patricia Diana Prasetiyo, Caesar Khairul Wallad, Timotius Ivan Hariyanto

**Affiliations:** a Department of Urology, Faculty of Medicine, Pelita Harapan University, Tangerang, Indonesia; b Department of Pathology, Faculty of Medicine, Pelita Harapan University, Banten, Indonesia; c Department of Urology, Fatmawati General Hospital, Jakarta, Indonesia; d Faculty of Medicine, Pelita Harapan University, Tangerang, Indonesia

**Keywords:** Vitamin D, fertility, reproductive biology, sperm, management

## Abstract

**Objective:**

Vitamin D has been hypothesized to have a potential role in altering sperm motility and metabolism. However, experimental studies have demonstrated inconsistent results between vitamin D and sperm parameters. This study aims to investigate the role of vitamin D supplementation to improve sperm parameters in infertile men.

**Methods:**

This is a systematic review and meta-analysis study. We comprehensively conducted a search on ClinicalTrials.gov, IRCT.ir, Europe PMC, and PubMed and collected published studies on vitamin D supplementation and sperm parameters for infertile men. The risk of bias was assessed by using Risk of Bias version 2 (RoB v2) and the statistical analysis was performed by using Review Manager 5.4 software.

**Results:**

Five trials with a total of 648 infertile men were included. Our meta-analysis showed that supplementation with vitamin D may significantly improve total sperm motility [mean difference 4.96 (95% CI 0.38, 9.54), *p* = 0.03, *I*^2^ = 69%], progressive sperm motility [mean difference 4.14 (95% CI 0.25, 8.02), *p* = 0.04, *I*^2^ = 89%], and normal sperm morphology [mean difference 0.44 (95% CI 0.30, 0.57), *p* < 0.00001, *I*^2^ = 0%] better than placebo in infertile men. However, total sperm count (*p = *0.15), sperm concentration (*p = *0.82), and semen volume (*p = *0.83) did not differ significantly between two groups.

**Conclusions:**

Vitamin D supplementation may improve sperm motility, progressive sperm motility, and morphology in infertile men. Vitamin D supplementation may be considered in managing male fertility issue.

## Introduction

Globally, infertility has become an important problem that affects medical, psychological, economic and social aspects [1]. World Health Organization (2010) defined infertility as an inability to conceive after ≥12 months of regular and unprotected sexual intercourse, and this condition affects approximately 15% of couples worldwide [2,3]. 40–50% of infertility cases are contributed by male factors, and causes may be related to congenital, acquired or idiopathic factors. Suboptimal sperm parameters were exhibited by approximately 2% of all men, and various studies supported a decline in sperm quality [1,4].

Semen analysis remains an initial evaluation of male fecundity [5]. The analysis is not directly measuring fertility, but any abnormalities in these parameters may correlate with fertility [6]. Semen analysis is complemented by a sperm functional assay, an indirect measures of spermatozoon’s biological capacity to reach and fertilize the ovum [6]. This assay may be categorized as test that indirectly assess sperm dysfunction through biochemical test (seminal oxidative stress and reactive oxygen species testing), bioassay of gamete interaction (sperm-zona pellucida binding test), acrosome reaction, sperm chromatin assay, and computer-aided semen analysis for sperm motion characteristics evaluation [6,7]. A meta-analysis of sperm function test demonstrated a high predictive power of sperm-zona pellucida binding assays and induced-acrosome reaction assays for fertilization outcome [7].

Research carried out during past decades attempts to investigate potential therapeutic approaches to improve male fertility, including sperm quality [8]. Previous literature shows that sperm quality is dependent on many parameters, including optimal levels of hormones and nutritional elements [9–11].

Recently, a surge in research on the possible health benefits of vitamin D has increased due to common deficiency in population [12]. Vitamin D is crucial for calcium and phosphate homeostasis regulation, and many non-classical actions in the human body [13]. Since VDR has been expressed in human testis, ejaculatory tract and human spermatozoa, many researches have been focused on identifying Vitamin D and VDR system in reproductive system [14]. Some studies have proposed Vitamin D role in altering sperm motility and metabolism, as well as driving sperm to undergo capacitation and acrosomal reaction, thus improving fertilization potential. A literature review analysing vitamin D role in seminal parameter showed that previous observational studies found controversial correlation between vitamin D status and sperm parameters [15]. Some authors observed a consistent positive correlation between vitamin D level and total sperm motility, progressive sperm motility, total sperm count and normal morphology [16–19]. Meanwhile, vitamin D was also reported to be unrelated to sperm count and morphology in other observational studies [20,21]. Experimental studies of vitamin D supplementation and sperm parameter also demonstrated an inconsistent association between [22–26]. Parameter of total sperm motility and progressive sperm motility were improved in Magshoumi and Padmapriya study, and total sperm number was also improved in Padmapriya study [25,26]. No other improvement of parameter were found in other studies.

Because of these inconsistencies, we aim to elaborate studies to establish its role among infertile men on their sperm parameter outcome, given that oral supplementation of vitamin D is economical and may confer benefits in many health outcomes.

## Materials and methods

### Eligibility criteria

We included randomized controlled trials that evaluated infertile/sub-fertile men (which may include oligozoospermia (the sperm concentration <15 × 10^6^/ml), asthenozoospermia (the motility of sperm <40% and rapid progressive sperm motility <32%), or teratozoospermia (<4% morphologically normal sperm cells)), which compared receiving vitamin D supplementation in any dosage with placebo or no treatment. We included studies that report at least one of the following outcomes: total sperm count, sperm concentration, semen volume, total sperm motility, progressive sperm motility, and normal sperm morphology;

Articles will be excluded if they have at least one of the exclusion criteria: articles focusing in infertile women; non-English language publications; non-randomized studies; articles which did not include control group; and unpublished articles.

### Search strategy and study selection

Systematic searching was done from four databases (ClinicalTrials.gov, Europe PMC, PubMed, and IRCT.ir). The following search terms were used: ‘(vitamin D OR cholecalciferol OR calcidiol OR calciferol OR calciol) AND (sperm OR spermatozoa OR spermatozoon OR semen OR male reproduction OR male reproductive) AND (infertile OR subfertile OR oligozoospermia OR asthenozoospermia OR teratozoospermia) AND (male OR man OR men) AND (trial OR clinical trial OR randomized clinical trial OR RCT)’ to filter studies from the year of 2010 through July 7th, 2022. The search was confined to English language studies. Two reviewers independently identified the eligible articles by titles and abstracts screening. References list were also evaluated to find relevant and potential studies. Duplicate articles would be removed. Finally, the same reviewers screened the full-text articles independently, and any discrepancies were resolved through discussion. Our study employed Preferred Reporting Items for Systematic Reviews and Meta-Analyses (PRISMA) guidelines [27,28].

### Data extraction and quality assessment

Two authors extracted data of authors, year of study, study design, sample size, age, vitamin D dosage, placebo used, vitamin D adverse events, number of patients in the intervention and control group, and outcome measures.

Two authors independently assessed the risk of bias and quality of the included studies by utilizing Risk of Bias version 2 (RoB v2) from Cochrane Collaborations [29]. This tool consists of five domains for methodological evaluation which include process of randomization; intended interventions deviations; missing outcome data; outcome measurement; and reported results selection. The randomized clinical trial (RCT) was classified as low risk of bias (low risk of bias for all domains), high risk (high risk of bias for ≥1 domains) or unclear risk (unclear risk of bias for ≥1 key domains).

### Statistical analysis

We used Review Manager 5.4 (Cochrane Collaboration) software to perform statistical analysis. We calculated standardized mean difference (SMD) and its standard deviations (SD) for the continuous variable outcomes using inverse variance method with random-effect models regardless of heterogeneity. Study heterogeneity was assessed by calculating *I*-squared (*I*^2^; inconsistency) and high degree of heterogeneity is considered when *I*^2^ statistic >50% [30]. Extrapolation of mean and standard deviations using formula of Wan et al. would be done when reported data were medians and interquartile ranges or medians and minimum-to-maximum ranges [31]. Funnel plot was constructed to assess the qualitative risk of publication bias.

## Results

### Study selection and study characteristic

**Figure 1.**
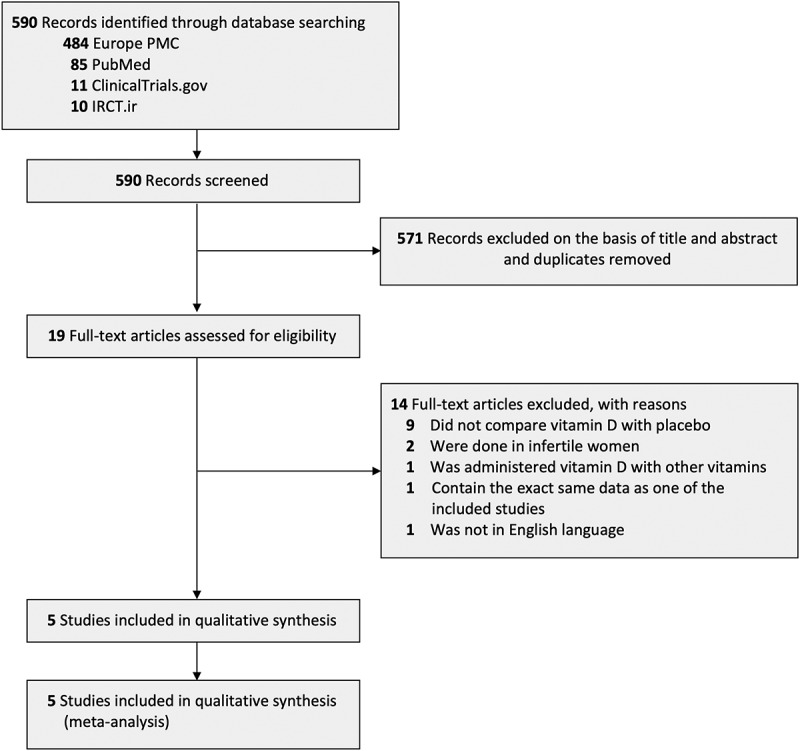
PRISMA diagram of the detailed process of selection of studies for inclusion in the systematic review and meta-analysis.

Initial search retrieved 590 studies from four database. After screening titles and abstracts and excluding duplicates, 19 articles were eligible, but further excluded to 14 articles after full-text screening. Nine articles did not compare vitamin D with placebo, two articles were done in infertile women, one article was administering vitamin D along with other vitamins, one article contained the exact same data as one of the included studies, and one article did not have full-text article in English-language. Five randomized clinical trial studies which included a total of 648 infertile/sub-fertile men were finally included in the analysis (Figure 1) [22–26]. Three of five studies were triple-blind RCT, and the remaining two studies were double-blind RCT. The range of sample sizes were 40–330. Infertility or sub-fertility of all samples included were assessed according to WHO criteria and were diagnosed by urologist. The dose for vitamin D supplementation used in included studies were ranged from 1,400 IU/day to 50,000 IU/week, with a total duration of 72 –150 days. Table 1 shows the characteristics of each included trials.

**Table 1.** Characteristics of included studies.

Study	Sample size	Design	Participants	Age (years)	Vitamin D dose	Placebo	Vit D adverse events	Outcomes^a^
Amini et al.^[22]^ (2020)	72	Triple-blind RCT	Infertile men aged 20–45 years old who undergone treatment in fertility clinic	34.6 ± 4.7	Vitamin D3 5000 IU/week for 8 weeks, followed by maintenance dose 5000 IU/month for 4 weeks	Oral paraffin	None	1,2,3,4,5,6
Gheflati et al.^[^^23]^ (2021)	40	Double-blind RCT	Men aged 18–45 years old diagnosed with asthenozoospermia by urologist	32.6 ± 1.3	Vitamin D3 50,000 IU/week for 8 weeks, followed by maintenance dose 50,000 IU/month for the remaining 4 weeks	Oral paraffin	None	1,3,4,5,6
Jensen et al.^[^^24]^ (2018)	330	Triple-blind RCT	Infertile men who had shown impaired semen quality according to WHO criteria	34.8 ± 6.6	Vitamin D3 300,000 IU dissolved in oil as initial dose, followed by tablets containing vitamin D3 1400 IU and calcium 500 mg once daily for 150 days	Oral paraffin	None	1,2,3,4,5,6
Maghsoumi-Norourazabad et al.^[^^25]^ (2022)	86	Triple-blind RCT	Infertile men (the motility of sperm <40% and rapid progressive sperm motility <32%) and vitamin D3 levels <30 ng/ml	34.7 ± 5.2	Vitamin D3 4000 IU/day for 3 months	Oral maltrodextrin	None	1,3,4,5,6
Padmapriya et al.^[^^26]^ (2022)	120	Double-blind RCT	Men diagnosed with OAT (oligoasthenoteratozoospermia) who attended the fertility OPD	33.9 ± 7.8	Vitamin D3 2000 IU twice a day for 72 days	Vitamin B complex supplementation	None	1,3,4,5

^a^Outcomes: 1=total sperm count; 2=sperm concentration; 3=semen volume; 4=total sperm motility; 5=progressive sperm motility; 6=normal sperm morphology.

### Quality assessment

The risk of bias assessment using ROB v2 is depicted in Table 2. All of the RCTs have low risk of bias in all five domains of methodological evaluation, therefore were deemed fit to be included in the meta-analysis.

**Table 2.** Risk of Bias version 2 (RoB v2) for assessment of clinical trial studies.

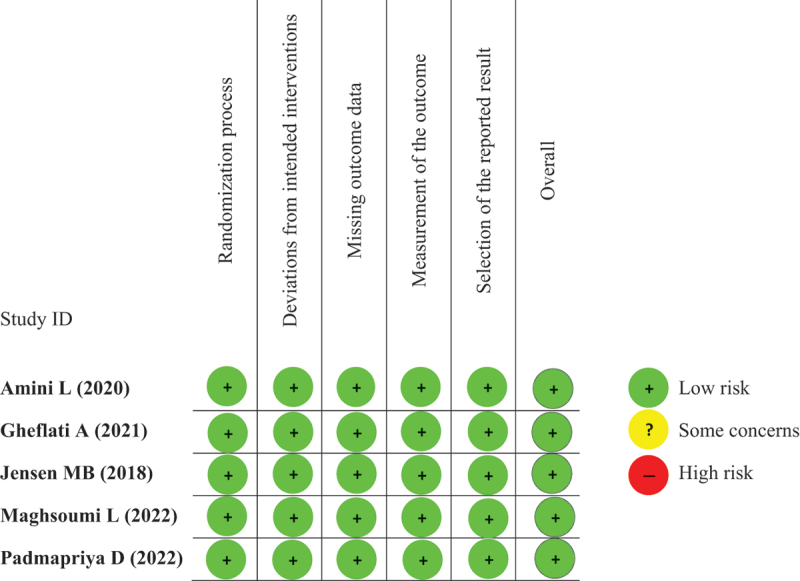

### Vitamin D supplementation and sperm parameters in infertile/sub-fertile men

#### Total sperm count

**Figure 2.**
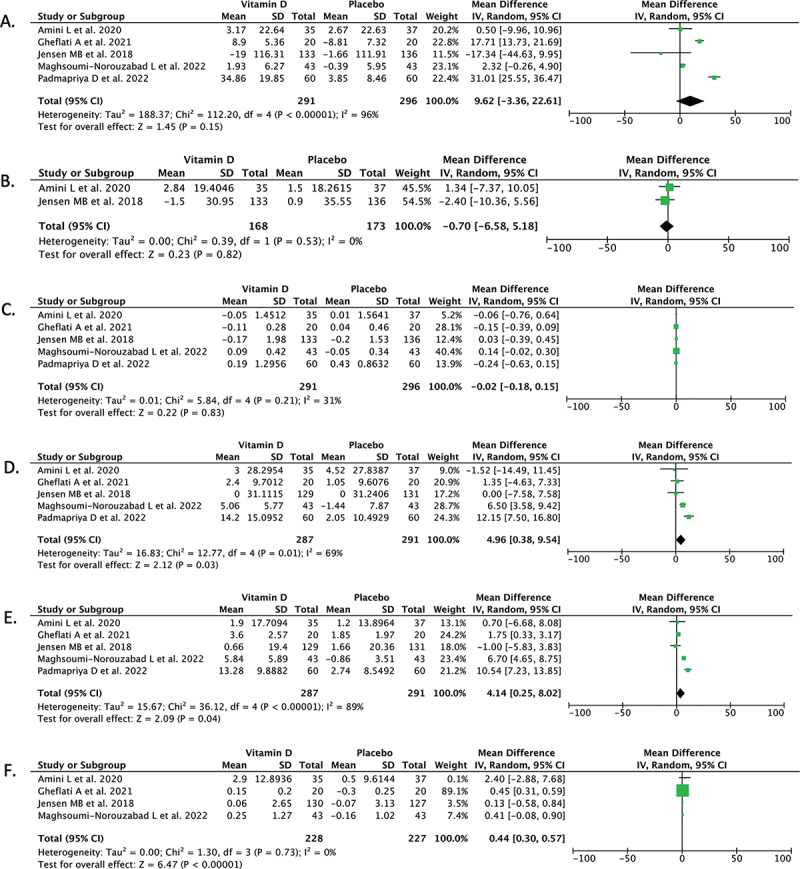
Forest plot that demonstrates the comparison between using vitamin D supplementation and placebo for men with infertility/sub-fertility in terms of total sperm count (A), sperm concentration (B), semen volume (C), total sperm motility (D), progressive sperm motility (E), and normal sperm morphology (F) outcomes.

Pooled analysis of five studies (*n* = 587) reporting on the total sperm count outcome showed that vitamin D supplementation did not offer any significant benefit in changing total sperm count when compared with placebo in men with infertility/sub-fertility [mean difference 9.62 (95% CI −3.36, 22.61), *p* = 0.15, *I*^2^ = 96%] (Figure 2A).

#### Sperm concentration

Pooled analysis of two studies (*n* = 341) reporting on the sperm concentration outcome showed that vitamin D supplementation did not offer any significant difference compared with placebo in changing the sperm concentration in men with infertility/sub-fertility [mean difference −0.70 (95% CI −6.58, 5.18), *p* = 0.82, *I*^2^ = 0%] (Figure 2b).

#### Semen volume

Pooled analysis of five studies (*n* = 587) reporting on the semen volume outcome showed that vitamin D supplementation did not offer any significant difference compared with placebo in changing semen volume in infertile/sub-fertile men [mean difference −0.02 (95% CI −0.18, 0.15), *p* = 0.83, *I*^2^ = 31%] (Figure 2c).

#### Total sperm motility

Pooled analysis of five studies (*n* = 578) reporting on the total sperm motility outcome showed that vitamin D supplementation was associated with a significant increase in the total sperm motility after treatment when compared with placebo in men with infertility/sub-fertility [mean difference 4.96 (95% CI 0.38, 9.54), *p* = 0.03, *I*^2^ = 69%] (Figure 2d).

#### Progressive sperm motility

Pooled analysis of five studies (*n* = 578) reporting on the progressive sperm motility showed that vitamin D supplementation was associated with significant increase in the amount of sperm which has progressive motility when compared with placebo in infertile/sub-fertile men [mean difference 4.14 (95% CI 0.25, 8.02), *p* = 0.04, *I*^2^ = 89%] (Figure 2e).

#### Normal sperm morphology

Pooled analysis of four studies (*n* = 455) reporting on the normal sperm morphology outcome showed that the changes in the amount of sperm which have normal morphology after treatments were significantly higher in the vitamin D group compared with the placebo in men with infertility/sub-fertility [mean difference 0.44 (95% CI 0.30, 0.57), *p* < 0.00001, *I*^2^ = 0%] (Figure 2f).

### Publication bias

**Figure 3.**
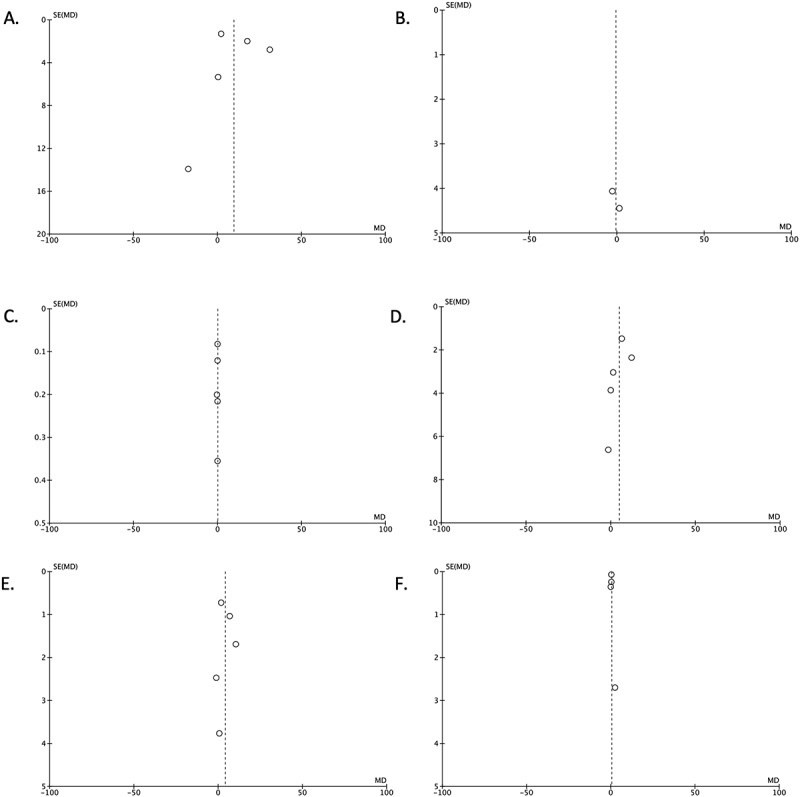
Funnel plot analysis for each outcomes of interest in this study: total sperm count (A), sperm concentration (B), semen volume (C), total sperm motility (D), progressive sperm motility (E), and normal sperm morphology (F).

Funnel plot analysis was relatively symmetrical inverted for all outcomes of interest in this study, suggesting that there was no publication bias (Figure 3a–f).

## Discussion

As far as our knowledge, this is the first meta-analysis study that assesses directly the vitamin D supplementation’s efficacy in improving the sperm parameters in infertile men through pooled results from RCTs. Our pooled analysis from five clinical trials have indicated that vitamin D supplementation was correlated to significant improvement in the sperm motility, progressive sperm motility and normal sperm morphology in infertile men when compared with placebo. However, vitamin D supplementation did not offer any significant benefit compared with placebo in terms of total sperm count, sperm concentration, and semen volume.

The results from this study were in alignment with the previous systematic review and meta-analysis study which has demonstrated that there was a significant association between serum 25(OH)D with sperm motility and sperm progressive motility [32]. However, this previous meta-analysis study has only linked the serum vitamin D concentrations with semen quality and did not assess directly the role of vitamin D supplementation, leaving a question whether supplementation with vitamin D can influence any of those parameters [32]. Our study may shed some lights into this matter by adding more evidence that vitamin D supplementation may directly improve sperm motility and progressive sperm motility. Moreover, this previous meta-analysis has combined studies with population of both fertile and infertile men, whereas our study only includes infertile men population to specifically assess the role of vitamin D supplementation in this population of interest. Our study also offers more solid evidence by including only RCTs which have higher evidence than observational studies and all were having low-risk of bias.

The association between vitamin D and improvement in sperm morphology has not been explained yet. Meanwhile, the improvement on sperm motility and progressive sperm motility seen with vitamin D supplementation could be explained by several mechanism. Sperm motility is a process which involves several complex signaling pathways and consumes adenosine triphosphate (ATP) for providing energy. Sperm motility is directly dependent on the energy availability acquired from ATP hydrolysis [33,34]. Mitochondria around the axoneme of sperm provides ATP as energy for sperm motility [35]. Through the cyclic adenosine monophosphate (cAMP)/protein-kinase A signaling pathway, 1,25(OH)_2_D (calcitriol) may play a role in phosphorylating mitochondrial electron chain complexes I and IV and ATP synthase inhibitor 1 [36,37]. This process will further regulate the respiratory chain complex protein function, enhance the oxidative phosphorylation function, and promote the ATP production with the end result of sperm motility enhancement [36,37]. 1,25(OH)_2_D may also bind to and activates vitamin D receptor (VDR) in the spermatozoa’s neck region, exerting its nongenomic effects, by activating phospholipase C and generating inositol triphosphate (IP_3_) production, subsequently opening the IP_3_-receptor (IP_3_R)-gated calcium channel in the redundant nuclear envelope and increasing the intracellular calcium (Ca^2+^) concentration [14,38,39]. The increase in intracellular calcium may upregulate the ATP production through enhancing the supply of nicotinamide adenine dinucleotide + hydrogen (NADH) to the respiratory chain complex that delivers hydrogen atoms to be utilized in oxidative phosphorylation [40]. Both cAMP and calcium have also been known as regulators of flagellar motility, therefore the increase in cAMP and calcium concentrations may also improve sperm motility through upregulation of flagellar motility from spermatozoa [41,42].

There are some limitations noted in this study. First, this analysis is limited by a relatively small number of studies with small sample size since only limited number of data were currently available. Second, the other registered clinical trial studies about vitamin D supplementation and sperm parameters are still recruiting or have not been completed yet (NCT03167008, NCT03829943, NCT03864198). Third, significant heterogeneities were also identified in some outcomes of interest in the study. These may be caused by different vitamin D dose used in the included studies. Nevertheless, we still believe that the results from our systematic review and meta-analysis can provide further insight to the management of male infertility.

## Conclusions

Our systematic review and meta-analysis of five RCTs showed that vitamin D supplementation in infertile men may significantly improve sperm motility, progressive sperm motility, and normal sperm morphology rate. This study suggests that vitamin D supplementation can be considered in the management of fertility issue in men. Larger clinical trials with proper methodology are still needed to validate our study results.

## Data Availability

The authors confirm that the data supporting the findings of this study are available within the article [and/or] its supplementary materials.
